# Standardized Upstream Processing of Ovarian Cancer Surgical Specimens for TIL-Oriented Cellular Workflows

**DOI:** 10.3390/cancers18183027

**Published:** 2026-09-18

**Authors:** Anna Biernacka, Joanna Kacperczyk-Bartnik, Ewa Witkowska, Julia Foremniak, Mariusz Bidziński, Paweł Derlatka, Justyna Marynowska

**Affiliations:** 1 Department of Regenerative Medicine, Maria Skłodowska-Curie National Research Institute of Oncology, 02-781 Warsaw, Poland; anna.biernacka@nio.gov.pl (A.B.); ewa.witkowska@nio.gov.pl (E.W.); julia.foremniak@nio.gov.pl (J.F.); 2 Department of Gynecologic Oncology, Maria Skłodowska-Curie National Research Institute of Oncology, 02-781 Warsaw, Poland; joanna.kacperczyk-bartnik@nio.gov.pl (J.K.-B.); mariusz.bidzinski@nio.gov.pl (M.B.); pawel.derlatka@nio.gov.pl (P.D.); 3 II Department of Obstetrics, Gynaecology and Gynaecological Oncology, Medical University of Warsaw, 02-097 Warsaw, Poland

**Keywords:** ovarian cancer, high-grade serous ovarian cancer, tumor-infiltrating lymphocytes, tissue processing, gentleMACS, cell viability

## Abstract

Background: Fresh ovarian cancer tissue is a demanding starting material for cellular workflows, particularly when the aim is to obtain tumor-derived suspensions suitable for further TIL-oriented procedures. In this study, we evaluated post-isolation cell concentration and viability in ovarian cancer specimens processed according to a standardized mechanical–enzymatic protocol. Methods: Biological material was collected from 24 patients undergoing routine surgery for ovarian cancer or suspected advanced ovarian malignancy. After macroscopic assessment and exclusion of extensively necrotic tissue, 19 specimens were included in the final analysis. The cohort was dominated by high-grade serous ovarian carcinoma, which accounted for 15 of 19 analyzed cases. Tissue specimens varied markedly in mass, with a median processed tissue weight of 2.05 g and a range from 0.11 to 14.93 g. Results: The median final cell concentration was 8.10 × 10^6^ cells/mL, with values ranging from 6.40 × 10^3^ to 9.26 × 10^8^ cells/mL. Median viability was 80.8%, although individual results ranged widely from 2.3% to 98.7%. The calculated median concentration of viable cells was 6.17 × 10^6^ viable cells/mL. Tissue mass showed a moderate positive association with final cell concentration (Spearman’s rho = 0.50, *p* = 0.028), but not with viability. Conclusions: These findings show that standardized processing of ovarian cancer surgical specimens can generate cell suspensions suitable for post-isolation quality assessment, even when the input material is highly variable. The present study focused on early post-isolation quality-control readouts obtained after tumor dissociation and did not evaluate TIL expansion capacity, detailed TIL phenotype, or antitumor function. The wide range of final cell concentrations underlines the biological and practical heterogeneity of ovarian cancer tissue and supports the need for careful reporting of early tissue-processing parameters in TIL-oriented workflows.

## Simple Summary

Immunotherapies adopting patients’ own immune cells recovered from within tumor tissue are under active investigation in ovarian cancer. Before such a therapy can be attempted, tissue obtained during surgery must first be converted into a suspension of individual living cells, and the quality of this initial step influences every subsequent stage of the process. In the present study, we analyzed tumor tissue from nineteen patients treated surgically for ovarian cancer, all processed according to a single standardized laboratory protocol, and assessed the number of cells recovered and the proportion that remained viable. In most cases, the majority of cells in the resulting suspension were viable; cell yield, however, differed substantially between patients, and larger tissue samples generally produced higher cell numbers. These results demonstrate that ovarian tumor tissue remains highly variable even under uniform handling and emphasize the importance of reporting these early processing parameters.

## 1. Introduction

Workflows oriented on tumor-infiltrating lymphocytes (TILs) require tumor-derived cell suspensions with acceptable cell viability that can be further used for immunophenotyping, selection, culture, or expansion [1,2]. Although attention is usually given to the later stages of TIL culture and product characterization, the initial processing of resected tumor tissue is a critical pre-analytical step; thus, the quality of the obtained material and the way in which the tissue is further processed into a single-cell suspension may influence the feasibility and reliability of downstream cellular procedures [3,4,5].

Ovarian cancer represents a challenging tissue source for this purpose. High-grade serous ovarian carcinoma (HGSOC) is the dominant and most aggressive subtype of epithelial ovarian cancer and is commonly diagnosed at an advanced stage [6,7]. At the molecular level, HGSOC is characterized by complex genomic instability, TP53 alterations observed in almost all cases, and considerable variability among patients [8,9]. From a practical laboratory perspective, ovarian tumor specimens may also differ in tissue mass, stromal content, hemorrhagic areas, necrosis, inflammatory infiltration, and the proportion of viable tumor tissue [10]. These specimen-related features may influence the composition and quality of the final cell suspension, particularly when solid tumor tissue is processed for downstream cellular analyses [11,12,13,14,15,16].

The presence of intratumoral T cells has prognostic significance in epithelial ovarian cancer [16,17,18]. However, the immune context of ovarian cancer is more complex than T-cell infiltration alone. Recent reviews emphasize that the ovarian cancer immune microenvironment includes multiple interacting immune and stromal compartments, including T cells, regulatory T cells, macrophages, myeloid-derived suppressor cells, NK cells, dendritic cells, cancer-associated fibroblasts, and endothelial cells. This complex and frequently immunosuppressive microenvironment contributes to the immunologically “cold” character of ovarian cancer and may partly explain the limited efficacy of several immunotherapeutic approaches in this disease [19]. In parallel, spatial and multi-omics studies have shown that T-cell infiltration and immune composition in HGSOC may differ between tumor sites and between distinct regions of the disease process [20,21,22,23]. Recent molecular studies also continue to identify regulatory networks associated with ovarian cancer progression and metastasis. For example, Huang et al. described a SOX4/AC093895.1/miR-1253/SOX4 feedback loop involved in ovarian cancer cell survival, migration, invasion, and metastatic potential [24]. Together, these data support the concept that ovarian cancer specimens represent biologically heterogeneous material at both the immune cell and molecular levels. Therefore, variability in the tumor specimen itself may remain an important determinant of the final cell suspension even when the laboratory procedure is standardized.

Preparation of single-cell suspensions from solid tumors can affect cell viability, the proportion of cell subsets, the amount of residual tissue fragments and aggregates, and the preservation of surface antigens used for downstream flow cytometric analyses. These parameters depend on the dissociation conditions; therefore, the choice of method is crucial for reliable tissue preparation [11,15]. Experimental studies have shown that mechanical and enzymatic tissue disaggregation may influence the phenotype and function of tumor-infiltrating immune cells [12,13]. In addition, dissociation conditions, including enzymatic digestion and temperature, can alter transcriptional profiles in single-cell analyses [14,25].

The aim of this publication was to evaluate the final post-processing cell concentration and viability in ovarian cancer surgical specimens processed according to a standardized mechanical–enzymatic tissue dissociation procedure. The study focuses on a real-world cohort dominated by HGSOC and emphasizes the variability of tumor material as a key factor influencing the characteristics of the final cell suspension.

## 2. Materials and Methods

### 2.1. Description of Study Group and Collection of the Samples

Initially, this study included biological material obtained from ovarian cancer specimens collected from 24 patients who underwent routine surgical treatment at the Department of Gynecologic Oncology, Maria Skłodowska-Curie National Research Institute of Oncology—National Research Institute. The patients were 41–74 years old at the time of surgery, with a mean age of 59.4 years and a median age of 61.5 years. The study group was dominated by patients with high-grade serous ovarian carcinoma (HGSOC), which is consistent with the clinical profile of patients undergoing surgery for advanced epithelial ovarian cancer [6,7,8]. Most patients presented with advanced-stage disease. FIGO stage IIIC was the most frequent clinical stage and was identified in 13 cases. This stage reflects macroscopic peritoneal disease outside the pelvis and/or regional lymph node involvement, in the absence of distant metastases. The remaining cases included FIGO stages IIIA1, IIIB, IIB, and IVB. One patient was diagnosed with disseminated malignancy most likely originating from the gastrointestinal tract; therefore, this case did not meet the FIGO classification criteria for ovarian cancer and was excluded from the ovarian cancer-specific FIGO interpretation. This case was also excluded from the entire quantitative analysis of cell concentration and viability, as it did not originate from an ovarian primary tumor.

Tumors were collected aseptically during cytoreductive surgery in the operating room. Only ovarian tumor tissue was collected and analyzed for the purpose of this study; peritoneal implants, omental tissue, and lymph nodes, even when resected simultaneously during the same cytoreductive procedure, were not included in the analyzed material. Subsequently, they were transported and stored in a dedicated MACS Tissue Storage Solution (Miltenyi Biotec, Bergisch Gladbach, Germany, Cat. No. 130-100-008) and then submitted for isolation on the day of collection or, at the latest, the following day. This storage medium and time window were selected based on manufacturer validation data demonstrating preserved cell viability (>85%), TIL composition, and activation status of tumor-infiltrating leukocytes after 24 and 48 h of storage at 2–8 °C in a murine tumor model [MACS Tissue Storage Solution application note]. Following macroscopic assessment for the presence of extensive necrotic areas, tissue material obtained from four patients was excluded from further processing and discarded according to institutional procedures, consequently narrowing the study group to 19 samples. Together with the case of non-ovarian origin described above, a total of five patients were excluded from the initial cohort of 24, yielding a final analyzed group of 19 samples.

### 2.2. Isolation Process on the gentleMACS Platform

During processing, macroscopically visible necrotic areas, adipose tissue, blood vessel networks, connective tissue, and other non-tumor components were mechanically removed using sterile instruments. After this preparation, the tumor specimen was measured with a ruler, weighed, and qualified for dissociation; all measurement results were recorded. The tissue was then mechanically minced into fragments approximately 1–2 mm in diameter and suspended in the dedicated medium.

Mechanical–enzymatic dissociation was performed on the gentleMACS platform using the Tumor Dissociation Kit, human (Miltenyi Biotec, Bergisch Gladbach, Germany, Cat. No. 130-095-929). Dissociation tubes were prepared according to the amount of processed tissue, using approximately one gentleMACS C Tube per 1 g of tumor material. For each C Tube, 4.7 mL of room-temperature Dulbecco’s Modified Eagle Medium + GlutaMAX (DMEM + GlutaMAX; Gibco, Paisley, UK, Cat. No. 31966-021) was added, followed by the prepared tumor fragments and the enzymatic components of the Tumor Dissociation Kit. According to the manufacturer’s protocol for 0.2–1.0 g of tumor tissue, the standard enzyme mixture contains 200 µL Enzyme H, 100 µL Enzyme R, and 25 µL Enzyme A. To preserve lymphocyte surface epitopes for anticipated TIL-oriented downstream applications, the amount of Enzyme R was reduced to approximately 20% of the manufacturer’s standard recommendation, corresponding to 20 µL per C Tube, in accordance with the kit guidance for the analysis of tumor-infiltrating leukocytes.

The C Tubes were closed tightly, placed in the gentleMACS adapter, and processed with heating using the TDK_1 program. Automated dissociation consisted of enzymatic incubation for 45 min at 37 °C with simultaneous programmed mechanical disruption and continuous automated mixing. After completion of the gentleMACS program, the resulting suspension was transferred through a 70-µm SmartStrainer cell strainer (Miltenyi Biotec, Bergisch Gladbach, Germany, Cat. No. 130-098-462) placed in a 50 mL Falcon tube. Before filtration, the strainer was pre-wetted with 1.5–2 mL DMEM + GlutaMAX supplemented with albumin. The strainer was subsequently rinsed with the same albumin-containing medium to recover the remaining cell suspension.

The filtered suspension was centrifuged for 5 min at 300× *g* at 20 °C. After removal of the supernatant, the cell pellet was resuspended in 1 mL PBS, followed by the addition of 9 mL of freshly prepared working Red Blood Cell Lysis Solution (Miltenyi Biotec, Bergisch Gladbach, Germany, Cat. No. 130-094-183). The suspension was vortexed and incubated for 2 min. After erythrocyte lysis, the suspension was centrifuged for 10 min at 300× *g* at 20 °C. The supernatant was discarded, and the final cell pellet was resuspended in PBS or culture medium and transferred for post-isolation quality control.

Albumin was incorporated as a fixed protein-containing component of the post-digestion processing step. The albumin-containing medium consisted of DMEM + GlutaMAX supplemented with Alburex 20, an albumin solution at a concentration of 200 g/L (Alburex 20, Marburg, Germany, Cat. No. 27007212763280). Protein-containing buffers are commonly used in enzymatic tissue dissociation workflows to reduce residual proteolytic activity and support subsequent handling of cell suspensions; however, the independent effect of albumin was not evaluated in the present study [26].

### 2.3. Quality Control of Dissociation Products

After centrifugation and final resuspension in medium, flow cytometry was performed to assess cell viability and cell count. Cell concentration, viability, and basic immune-cell composition were assessed by automated flow cytometric analysis using the MACSQuant^®^ Analyzer 16 Flow Cytometer (Miltenyi Biotec, Bergisch Gladbach, Germany). Measurements were performed using the Miltenyi Biotec-tested panel 35 (MBTP 35; Miltenyi Biotec, Bergisch Gladbach, Germany, Cat. No. 130-120-640). Cell viability was determined using 7-AAD Staining Solution (Miltenyi Biotec, Bergisch Gladbach, Germany, Cat. No. 130-111-568), with 7-AAD-negative events considered viable.

Prior to testing of each sample, instrument quality control was performed using MACSQuant^®^ Calibration Beads (Miltenyi Biotec, Bergisch Gladbach, Germany) in accordance with the manufacturer’s protocol and internal laboratory procedures. Events were selected based on forward scatter and side scatter characteristics to exclude debris and non-cellular particles. Where applicable, doublets and aggregates were excluded using scatter-based gating. The CD45-positive fraction was identified as the leukocyte-derived compartment within the tumor-derived suspension. CD3-positive T cells were assessed within the CD45-positive fraction, and CD4-positive and CD8-positive subsets were assessed within CD3-positive T cells.

The analyses were performed on the CD45+ cell population, which included cells present in the tumor material and constituted the population of cells infiltrating the tumor. Cell concentration was expressed as the number of cells per 1 milliliter of the final suspension. The concentration of live cells was calculated as the product of the final cell concentration and the viability fraction.

**Figure 1:**
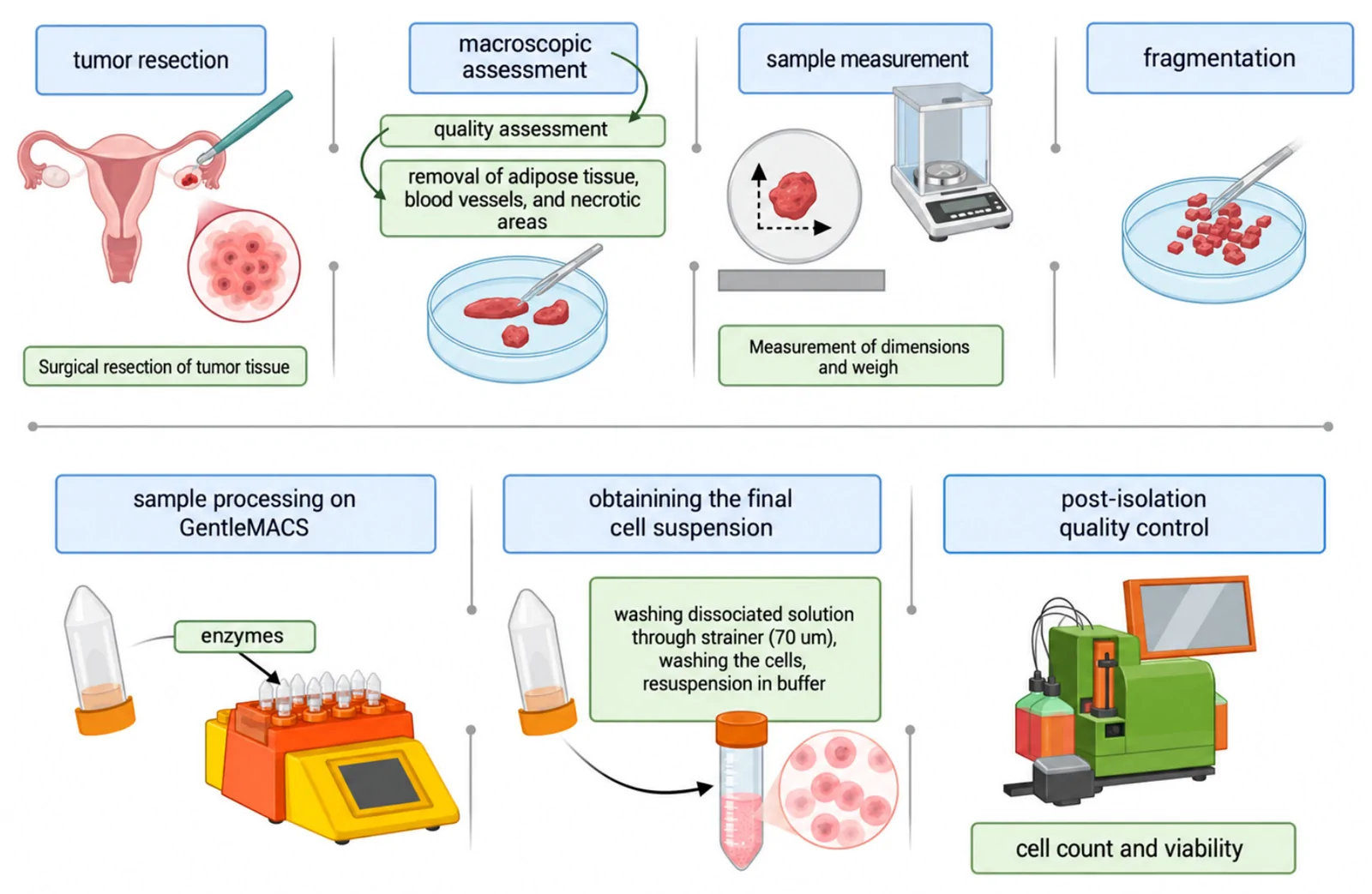
Overview of the standardized workflow for ovarian tumor tissue processing and post-isolation quality control.

Every step of the described process is pictured in Figure 1.

## 3. Results

A total of 19 samples from patients who underwent surgery for ovarian cancer or suspected advanced ovarian cancer were analyzed. The patients’ ages ranged from 41 to 74 years, with a median of 62 years. The predominant histological diagnosis in the study group was high-grade serous ovarian carcinoma (HGSOC), which was identified in 15 out of 19 cases (78.9%). The remaining cases involved other or indeterminate histological types and constituted a minority of the analyzed group.

**Figure 2:**
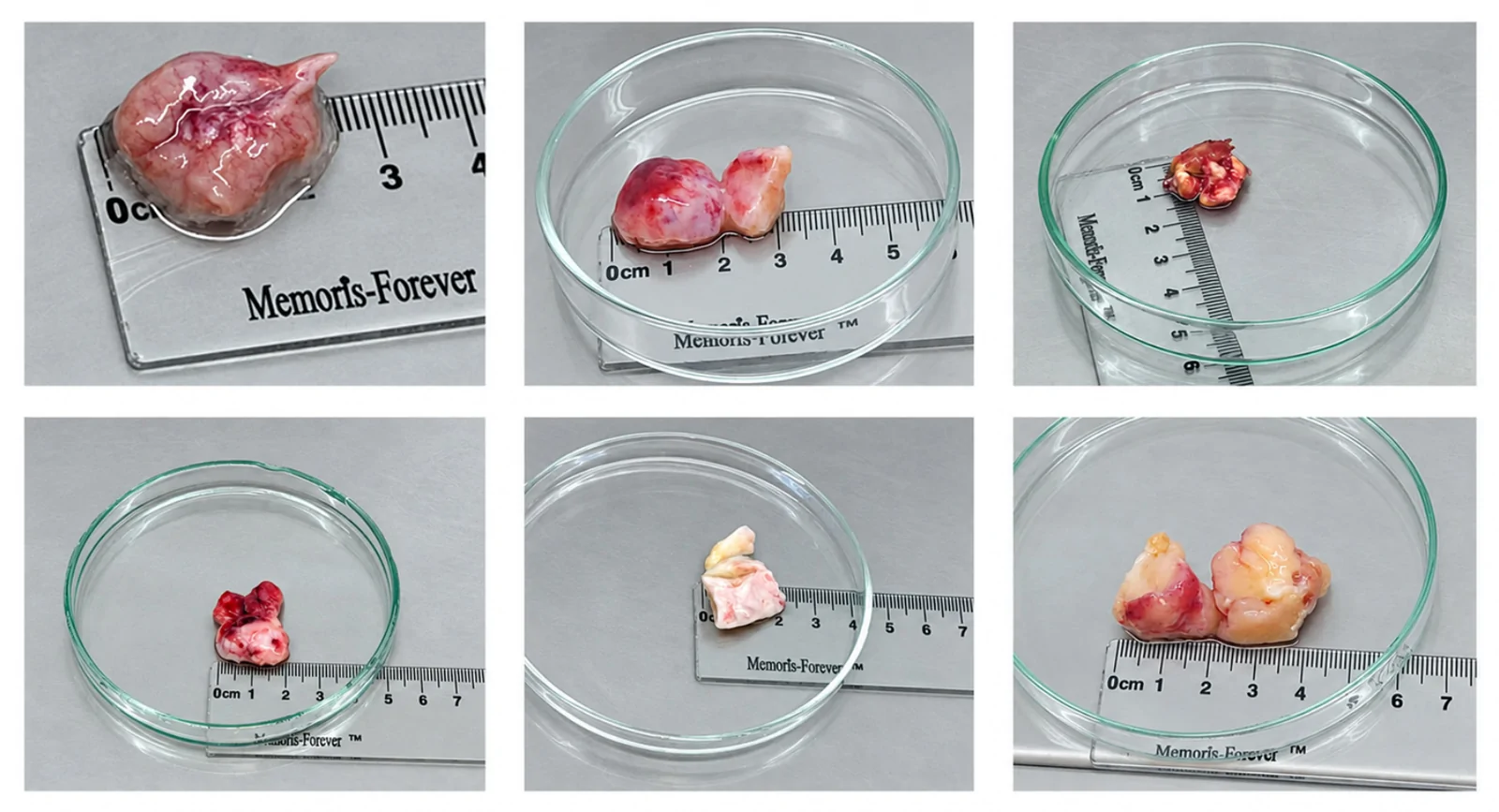
Pictures of six representative ovarian tumor specimens during the procedure.

The analyzed samples were characterized by significant variation in the weight of the processed tumor tissue. The median mass of the obtained tissue was 2.05 g, with the smallest analyzed sample weighing 0.11 g and the largest 14.93 g. In the HGSOC subgroup, the median mass of the material was similar to that observed in the entire cohort and amounted to 1.90 g. The data indicate that the analyzed material included both very small tumor fragments and samples many times larger in mass, reflecting the high variability of the surgical material available for further laboratory processing. In Figure 2, we present six representative photographs of tumor specimens during measurements.

**Figure 3:**
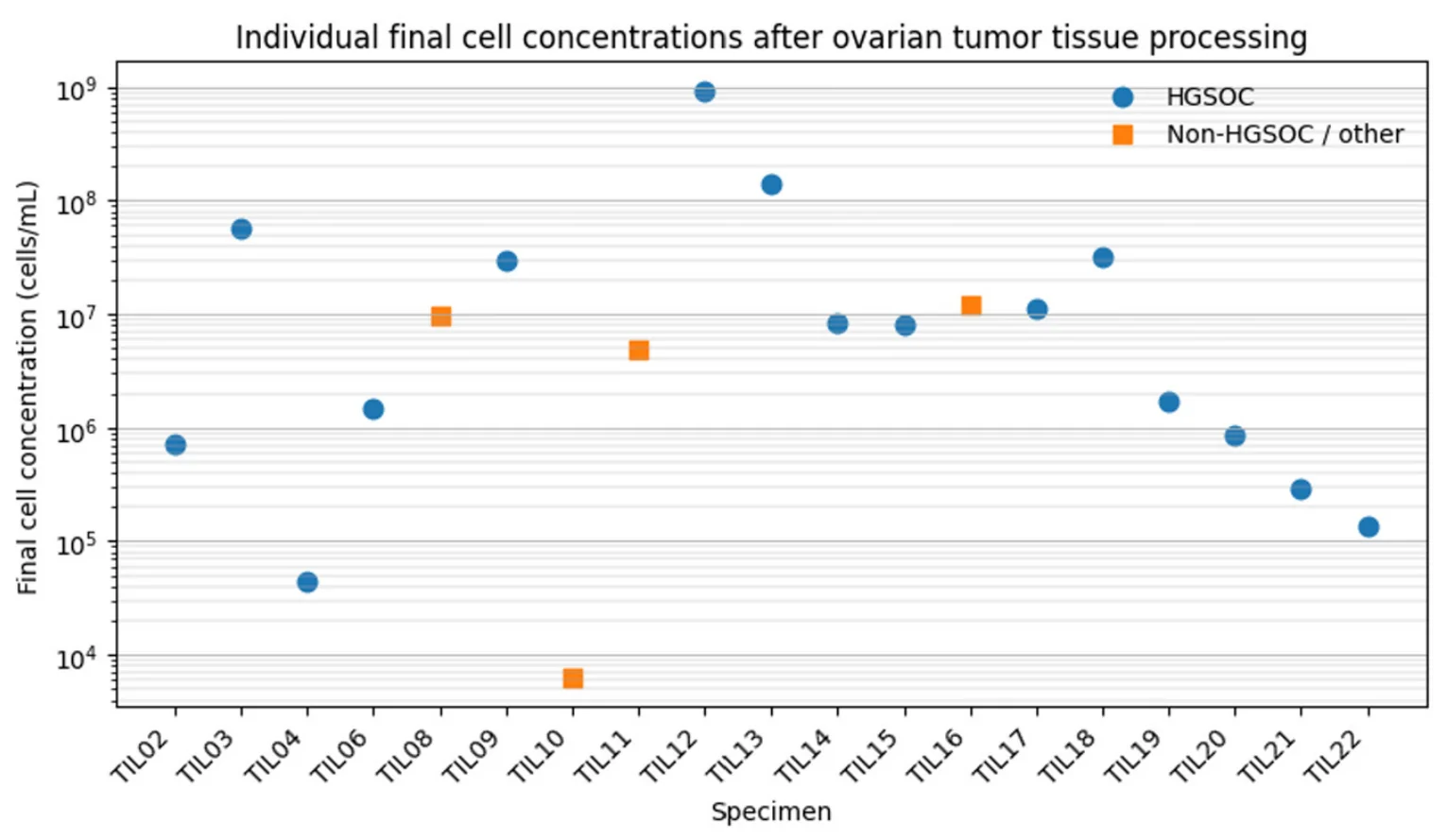
Individual final cell concentrations after standardized mechanical–enzymatic processing of ovarian cancer surgical specimens. Each point represents one analyzed specimen. The y-axis is presented on a logarithmic scale because final cell concentrations spanned more than five orders of magnitude. HGSOC cases are indicated separately from non-HGSOC or other/indeterminate histological types.

The final cell concentration showed substantial inter-sample variability and a markedly asymmetric distribution. In the entire cohort, the median concentration was 8.10 × 10^6^ cells/mL, with values ranging from 6.40 × 10^3^ to 9.26 × 10^8^ cells/mL. The mean concentration was 6.55 × 10^7^ cells/mL, substantially exceeding the median, consistent with a right-skewed distribution. Overall, the observed concentrations spanned more than five orders of magnitude. In the HGSOC subgroup, the median final cell concentration was 8.10 × 10^6^ cells/mL, with a range of 4.50 × 10^4^ to 9.26 × 10^8^ cells/mL. Because of the pronounced asymmetry of the distribution, individual specimen-level values are presented on a logarithmic scale in Figure 3. Due to the limited number of non-HGSOC and other/indeterminate cases, these subgroups were summarized descriptively and were not subjected to formal statistical comparison.

The viability of the final suspension after dissociation also showed significant variability, with results ranging from 2.3% to 98.7%; the median viability in the study group was 80.8%. In the HGSOC subgroup, the median viability was comparable at 80.6%. These results indicate that, despite high variability among individual samples, most of the analyzed suspensions were characterized by preserved cell viability. Table 1 summarizes the results for the entire study group.

Basic immunophenotyping data from the initial post-isolation flow cytometric assessment were available. CD45-positive leukocytes were identified in all available records, with a median of 19.65% of live cells (range, 1.71–91.04%). CD3-positive T cells represented a median of 65.96% of CD45-positive cells (range, 7.17–85.14%). CD4-positive and CD8-positive T-cell subsets showed inter-sample variability, with median values of 13.54% and 26.09% of CD3-positive cells, respectively. The distribution of the main immune-cell parameters is summarized in Supplementary Table S1. These data were used as basic post-isolation characterization of the available samples and were not interpreted as a measure of TIL expansion capacity or antitumor function.

In summary, the study group consisted mainly of HGSOC cases and was characterized by significant heterogeneity in terms of the mass of tumor material obtained for the subsequent isolation procedure. The final cell concentration and the concentration of live cells per milliliter showed a wide range of values, while the median viability remained high in both the entire study group and the HGSOC subgroup.

## 4. Discussion

This study evaluated the final quantitative and qualitative parameters of cell suspensions obtained from ovarian cancer tissue processed according to a standardized, internally validated mechanical–enzymatic isolation procedure. The most important observation was the noticeable variability in the final cell concentration and the concentration of live cells per milliliter among samples, despite a relatively high median cell viability. In the study group, cases of tissue diagnosed as HGSOC predominated; however, the group remained heterogeneous in terms of the weight of tissue material obtained for further processing. The range of tumor weights included both small tissue fragments and samples weighing several grams, which reflects the real-world conditions of tissue collection in ovarian cancer during surgical resection of lesions. In this context, the moderate positive correlation between tissue weight and the final cell concentration (cells/mL) indicates that the amount of input material may partially influence the cell density of the final suspension but does not fully explain the variability observed among samples. The lack of a relationship between tissue weight and viability, however, suggests that the biological quality of the material does not depend solely on its quantity.

The observed variability in results is consistent with the current understanding of ovarian cancer biology, with particular emphasis on HGSOC. Despite its seemingly homogeneous histopathological classification, HGSOC is characterized by marked molecular and cellular heterogeneity [8,27,28,29], while spatial variability of the tumor-immune microenvironment has also been described in ovarian cancer and HGSOC [20,21,22,23]. Single-cell studies have confirmed that the heterogeneity of HGSOC extends not only to the phenotype of cancer cells but also to the tumor microenvironment, which includes connective tissue stromal cells and cells involved in the immune response [27,28,29]. Additionally, studies evaluating the spatial heterogeneity of the tumor microenvironment have shown that lymphocytic infiltrates and the type of immune responses can vary significantly between different tumor sites in the same patient and among patients with the same type of cancer [2,20,21,22,23]. In light of these data, the wide range of cellularity and viability observed in our material should not be regarded solely as technical inconsistency but rather as the result of the coexistence of biological, histological, and preanalytical variability. It should also be emphasized that the analyzed cohort was not histologically homogeneous: alongside the predominant HGSOC subtype (15/19 cases), it also included four cases of other or indeterminate histological types of epithelial ovarian cancer. This inter-subtype histological heterogeneity represents an additional, distinct source of biological variability, separate from the molecular and architectural heterogeneity described above within HGSOC itself, and may have contributed to the wide range of results observed across the entire analyzed group.

This interpretation is also consistent with recent literature emphasizing that ovarian cancer heterogeneity includes not only spatial variation in immune infiltration but also complex immune-suppressive interactions within the tumor microenvironment [19] and molecular regulatory networks associated with tumor progression and metastatic potential [24].

An important aspect of our study was that all samples were processed according to a single, internally standardized protocol, as the preparation stage of each cell suspension from a solid tumor can be a source of variability. The literature emphasizes that the method of tissue dissociation directly affects the viability of isolated cells, the cellular composition of the final suspension, and the presence of tissue debris and aggregates, as well as the preservation of surface antigens analyzed during flow cytometry assessment [11]. Grange et al. demonstrated the influence of different methods of mechanical and enzymatic dissociation of solid tumors on the phenotype and function of immunologically active tumor-infiltrating cells, indicating that the method used at the early stage of preparation can affect the detectability of selected surface markers [12]. Similarly, Quatromoni et al. noted that the protocol used for tumor tissue processing can alter the immunological composition of the resulting cell suspension and directly influence the observed surface marker expression profile. They concluded that a gentle, standardized mechanical–enzymatic approach may limit the negative impact of dissociation on the quality of the final product [13]. Furthermore, single-cell studies have shown that tumor dissociation conditions, such as temperature and the type of enzymatic digestion, can affect not only the quality parameters of the suspension but also the gene expression profile of individual cells [14].

Therefore, the use of a standardized, in-house developed procedure ought to be considered one of the most important elements of the study. Standardizing the criteria and methods for sample selection, processing, and evaluation minimizes technical variability and allows differences between samples to be interpreted primarily in terms of changeability of the clinical material. However, it is worth mentioning that the procedure has not been optimized relative to other methods. In this study, neither a parallel control nor an alternative isolation protocol was used; therefore, the results do not allow a conclusion regarding the superiority of our procedure compared to other tissue dissociation methods.

What warrants special attention is the inclusion of albumin as a standard component of the procedure. It served as an enzymatic activity inhibitor, all the while stabilizing the cell suspension after the digestion step. However, since samples processed without albumin were not analyzed, it is not possible to attribute an unambiguous and independent effect of albumin on viability, cell content, and concentration of live cells per milliliter. Albumin was a consistent component of an internally defined workflow that was applied uniformly to all samples. This approach follows the logic of methodological studies, in which the consistency of key process steps is intended to reduce technical variability but does not in itself prove the effectiveness of a single component used in the specific procedure.

Another practical aspect of standardization was the qualification of the material prior to isolation. Samples characterized by the presence of significant areas of necrosis were not qualified for subsequent stages of the isolation procedure. The presence of significant necrotic foci was the sole and key criterion for disqualifying the material, since the presence of extensive necrosis can increase the proportion of debris—that is, dead cells—which results in nonspecific background in the final suspension, thereby compromising its quality for further cellular analyses. In this study, the effect of the degree of necrosis on the final parameters was not assessed separately, as excessively necrotic material was not included in the procedure. This stage should therefore be regarded as a practical quality control of the input material rather than as an analysis of another biological variable. It should be explicitly noted that this exclusion criterion was based on qualitative macroscopic assessment, without a predefined quantitative threshold for the percentage of necrotic area or a minimum required dimension of viable tissue. Formalized clinical TIL manufacturing protocols (e.g., lifileucel) apply quantitative acceptance criteria, such as a minimum aggregate viable tissue diameter and a defined maximum percentage of necrosis; adopting comparable numerical thresholds represents a potential direction for further standardization of this step in future, more mature versions of the protocol.

A further methodological limitation concerns cold ischemia time. Tissue was transferred to MACS Tissue Storage Solution immediately after surgical excision and processed on the day of collection or, at the latest, the following day. Manufacturer validation data indicate that this medium preserves cell viability, TIL composition, and activation status for at least 24–48 h at 2–8 °C; however, this validation was performed in a murine tumor model rather than in human tissue, and the exact elapsed time was not recorded for each individual sample. Consequently, uncontrolled variability within this time window cannot be entirely excluded as a contributor to the observed variability in cell concentration and viability. Future studies should record the precise, hour-resolved interval between surgical excision and initiation of isolation as a covariate to empirically verify the applicability of this storage medium validation to human ovarian cancer tissue.

A further limitation concerns the feasibility of multivariable analysis. Given the small sample size (*n* = 19), the absence of variance in the necrosis variable within the analyzed cohort (all included samples were macroscopically free of extensive necrosis, as this was an exclusion criterion applied prior to processing), and the lack of individually recorded, hour-resolved processing-time data for each sample, a formal multivariable regression model adjusting the tissue mass-cell concentration association for histological subtype, necrosis, and processing time was not performed. The observed association between tissue mass and final cell concentration is therefore reported solely as a bivariate correlation (Spearman’s rho = 0.50, *p* = 0.028). Verification of this association in a multivariable model, incorporating histological subtype, degree of necrosis, and precisely measured processing time, represents an important direction for future, larger studies.

An additional limitation concerns the use of albumin during post-digestion processing. All samples in this study were processed using an identical albumin-containing buffer; consequently, no parallel comparison group processed without albumin was available, and the independent contribution of albumin to cell viability, cellular yield, or the concentration of live cells could not be quantitatively determined in this cohort. The stabilizing and residual-enzymatic-activity-limiting role attributed to albumin is therefore based on established literature regarding protein-containing buffers in enzymatic tissue dissociation workflows rather than on direct experimental verification in the present study. Future studies could evaluate this factor experimentally through a parallel comparison of protocols with and without albumin.

A further limitation is that short-term post-isolation stability (i.e., cell viability and recovery after several hours of culture) was not assessed. The final cell suspension obtained after isolation was directed in its entirety into a closed, Good Manufacturing Practice (GMP)-compliant TIL culture system; consequently, no additional aliquot could be withdrawn for stand-alone stability testing outside this closed system without compromising sterility and GMP compliance, nor at the expense of the material available for the intended culture. Such an assessment could be considered in future studies using surplus material or a dedicated validation cohort processed outside the main, clinical, GMP-compliant culture pathway.

The significance of the results obtained should be considered in the context of further cellular applications, such as procedures aimed at the production of TILs. In ovarian cancer, the presence of tumor-infiltrating lymphocytes has biological and clinical significance, and the association between the presence of T lymphocytes within the tumor and a better prognosis in patients with epithelial ovarian cancer has been well documented [16,17,18,30]. Owens et al. demonstrated that TILs capable of an immune response against cancer cells can be isolated from ovarian tumors and cultured in vitro [4]. Since our study did not assess TIL expansion, immunological phenotype, or antitumor activity of the isolated cells, its results should be interpreted strictly as an evaluation of the stage preceding TIL production.

It is worth noting that this study did not directly examine the cellular composition of the final suspension. The evaluated parameters—cell concentration, viability, and the calculated concentration of live cells—are qualitative and quantitative measures; they do not provide insight into the ratio of tumor cells, lymphocytes, stromal cells, myeloid cells, or other components of the tumor microenvironment. For this reason, the results should not be interpreted as data on TIL numbers nor as evidence of the effectiveness of isolating a specific immune cell population. They are useful in the context of describing and qualifying cellular material derived from ovarian cancer specimens for further processing.

A strength of the study is the use of actual surgical specimens processed according to a standardized procedure, as well as the consistent reporting of the final parameters of the cell suspension.

The results indicate that a standardized mechanical–enzymatic procedure for processing ovarian cancer tissue yields final cell suspensions that can be evaluated for cell count and viability. Furthermore, the wide range of values observed for the final cell concentration and the concentration of live cells per milliliter clearly highlights the significant heterogeneity of ovarian cancer tissue. In future studies, it will be appropriate to supplement this assessment with a direct characterization of cellular composition, an analysis of the impact of preanalytical variables, and an evaluation of the relationship between the quality of the input material and the results of subsequent TIL culture or expansion.

## 5. Conclusions

The results presented in this study indicate that the use of a standardized procedure for processing tumor material obtained from ovarian cancer patients during cytoreductive surgery allows for the production of viable cell suspensions after dissociation, despite considerable variation in the mass and characteristics of the source tissue. However, it should be clearly stated that the cell concentration and viability demonstrated in this study are only preliminary, quantitative indicators of the quality of the obtained material and are not sufficient to establish its suitability for further therapeutic procedures based on tumor-infiltrating lymphocytes (TILs). Future studies should therefore evaluate immune-cell recovery, detailed TIL phenotype, the expansion capacity of these cells, their functional activity (including anti-tumor potential), and the reproducibility of the results obtained in larger and more diverse populations of ovarian cancer patients [31].

## Figures and Tables

**Table 1:** Summary of the study group and the results of cell viability and concentration of the suspensions.

	Number
Samples analyzed	19
HGSOC in the study group	15/19 (78.9% of the entire study group)
Median weight of processed tissue	2.05 g
Median final cell concentration	8.10 × 10^6^ cells/mL
Median final viability	80.8%
Median concentration of live cells	6.17 × 10^6^ live cells/mL

## Data Availability

The raw data supporting the conclusions of this article will be made available by the authors upon request.

## Supplementary Materials

The following supporting information can be downloaded at https://www.mdpi.com/article/10.3390/cancers18183027/s1, Table S1: Basic MBTP35 immunophenotyping summary for available initial post-isolation records.
