# Supplementary material for: Standardized Upstream Processing of Ovarian Cancer Surgical Specimens for TIL-Oriented Cellular Workflows
Source: Cancers (Basel). 2026 Sep 18;18(18):3027. doi: 10.3390/cancers18183027 (PMC13605654; doi:10.3390/cancers18183027)
Supplement: Supplementary file 1 [file cancers-18-03027-s001.zip › cancers-4526946-supplementary.pdf]

Supplementary Table S1

Basic MBTP35 immunophenotyping summary for available initial post-isolation records

Values are presented as median, interquartile range (IQR), and range. The table summarizes available initial post-isolation records only.

Parameter	Definition	Median	Range	Unit	Comment
CD45+ leukocytes	Percent of live cells	19.65	1.71–91.04	%	Leukocyte-derived compartment within tumor-derived suspension
CD3+ T cells	Percent of CD45+ cells	65.96	7.17–85.14	%	Basic T-cell fraction within leukocyte compartment
CD4+ T cells	Percent of CD3+ cells	13.54	0.16–84.33	%	Basic CD4 T-cell subset
CD8+ T cells	Percent of CD3+ cells	26.09	0.01–73.13	%	Basic CD8 T-cell subset
CD45+ concentration	Corrected count per mL	1.92 × 10^5	2080–2570000	cells/mL	Concentration from MBTP35 acquisition; available subset only
CD3+ concentration	Corrected count per mL	8.18 × 10^4	855–1320000	cells/mL	Concentration from MBTP35 acquisition; available subset only

Abbreviations: MBTP 35, Miltenyi Biotec-tested panel 35; TIL, tumor-infiltrating lymphocyte.

These data are intended as basic post-isolation characterization of the available samples and should not be interpreted as TIL expansion capacity, antitumor activity, final product quality, or release criteria.
